# β-HHVs and HHV-8 in Lymphoproliferative Disorders

**DOI:** 10.4084/MJHID.2011.043

**Published:** 2011-10-24

**Authors:** C. Quadrelli, P. Barozzi, G. Riva, D. Vallerini, E. Zanetti, L. Potenza, F. Forghieri, M. Luppi

**Affiliations:** Section of Hematology. Department of Oncology, Hematology and Respiratory Diseases. Azienda Ospedaliero-Universitaria di Modena- Policlinico, Modena Italy

## Abstract

Similarly to Epstein-Barr virus (EBV), the human herpesvirus-8 (HHV-8) is a γ-herpesvirus, recently recognized to be associated with the occurrence of rare B cell lymphomas and atypical lymphoproliferations, especially in the human immunodeficiency virus (HIV) infected subjects. Moreover, the human herpesvirus-6 (HHV-6), a β-herpesvirus, has been shown to be implicated in some non-malignant lymph node proliferations, such as the Rosai Dorfman disease, and in a proportion of Hodgkin’s lymphoma cases. HHV-6 has a wide cellular tropism and it might play a role in the pathogenesis of a wide variety of human diseases, but given its ubiquity, disease associations are difficult to prove and its role in hematological malignancies is still controversial. The involvement of another β-herpesvirus, the human cytomegalovirus (HCMV), has not yet been proven in human cancer, even though recent findings have suggested its potential role in the development of CD4^+^ large granular lymphocyte (LGL) lymphocytosis. Here, we review the current knowledge on the pathogenetic role of HHV-8 and human β-herpesviruses in human lymphoproliferative disorders.

## Introduction

Epstein-Barr virus (EBV) is a γ-herpesvirus well recognized to be involved in the development of human B and NK/T cell lymphomas, either in the general population or in the immunosuppressed individuals. EBV is a lympho- and epitheliotropic γ-herpesvirus apparently carried as an harmless passenger in the immunocompetent host. Alterations in the delicate balance between the virus and the host immune control may result in a wide range of EBV-associated diseases: the simplest scenario is the outgrowth of EBV-transformed B-lymphoblasts, expressing the full array of EBV latent gene (EBNA1, 2, 3A, 3B, 3C, LP, LMP1 and LMP2) leading to the development of post-transplant lymphoproliferative disease (PTLD) in immunodeficient subjects. EBV is also associated to malignancies in immunocompetent hosts arising from either epithelial, T cell or B cell origin in which is present with a limited pattern of latency genes: latency II (expression of EBNA1, LMP2A) is typical of Nasopharingeal Carcinoma, Gastric Carcinoma and Hodgkin’s Lymphoma; latency I (expression of EBNA1) is associated to the Burkitt Lymphoma.^1^

In addition to EBV, another γ-herpesvirus, Kaposi’s sarcoma-associated herpesvirus (KSHV or HHV-8) is oncogenic. Among β-herpesviruses, several investigators have suggested that human herpesvirus-6 (HHV-6) also may be an oncogenic virus. Here, we review the current knowledge on the pathogenetic role of human β-herpesviruses and HHV-8 in human lymphoproliferative disorders.

## β HERPESVIRUSES

### HHV-6

#### Epidemiology and biology

HHV-6 was first isolated in 1986 and later two viral variants have been identified, namely HHV-6A and HHV-6B, showing an overall nucleotide sequence identity of 90%. HHV-6 is ubiquitous in human throughout the world, with seroconversion occurring early in life.^2^,^3^ Salivary contact is likely to be the vehicle for transmission, but intrauterine passage is also possible. HHV-6 can be transmitted by blood products and with bone marrow and solid organ transplantation. Through its cellular receptor CD46, an ubiquitary complement regulatory glycoprotein^4^, HHV-6 can primarily infect either early self-renewing bone marrow precursors or mature blood cells, as well as oropharinx/salivary glands, epithelial mucosa of female genital tract and brain tissue. Following primary infection, HHV-6 can persist lifelong mainly in monocytes and other peripheral blood mononuclear cells.^3^ Only rare cells remain latently infected in healthy individuals, as shown by PCR testing. Of note, HHV6, unique among all the herpesviruses, exhibits a particular form of persistence in the infected cell, consisting in the integration of the whole viral genome into host chromosomes. The prevalence of the ‘chromosomal integration of HHV-6’ (CIHHV-6) ranges from 0.2% to 3% among different geographical areas.^5^,^6^ It has been observed that the main route of acquisition of CIHHV-6 is the vertical transmission, which implies that at least one copy of viral DNA is present in all the nucleated cells of the host. The HHV-6 genome shows human telomere-like repeat sequences at both its ends and this may foster the viral integration in some preferred chromosomal regions (mainly 17p13.3, 22q13, 1q44), which are close to or within the telomeres.^7^–^10^

HHV-6 has been demonstrated to efficiently replicate in vitro and cause a cytopathic effect either in CD4+ T lymphocytes or in thymocytes, inducing a suppression of T-cell functions. Cells transfected with HHV-6 can cause tumors in nude mice.^11^ However, the evidence linking HHV-6 to human hematological malignancies is circumstantial, and far from definitive.^12^ HHV-6 DNA can transform human epidermal keratinocytes and NIH 3T3 cells in vitro. 13–14 HHV-6 has a number of unique genes that are plausible causes of oncogenesis. Its ORF-1 gene encodes a protein that is capable of transforming NIH 3T3 cells in vitro, and cells expressing ORF-1 protein produce fibrosarcomas when injected into nude mice.^15^ The ORF-1 protein appears to maintain the transformed state of tumor cells by binding p53 and thereby inhibiting its tumor suppressor properties.^16^ HHV-6 also has a unique immediate early gene called U95 that has binding sites for nuclear factor-kappa B (NF-kB).^17^ Dysregulation of NF-kB has been postulated to contribute to cancer, through its effects on both the proliferative and apoptotic pathways.^18^ (Table 1)

#### Hodgkin’s disease

Reports differ as to the possible role of HHV-6 in Hodgkin’s disease (HD). Torelli et al.^19^ reported finding HHV-6 sequences by PCR in 3 of 25 cases of HD, all nodular sclerosis type, and in none of 41 cases of non-Hodgkin’s lymphoma. Krueger et al.^20^ performed immunohistochemical studies of tumors from 103 patients with HD, and found tissue sections to be infected frequently by both EBV and HHV-6; lymphocytes and histiocytes were infected preferentially. Lacroix et al.^21^ found HHV-6 DNA more frequently in the nodular sclerosis form of HD: of 73 patients with nodular sclerosis, 39 (49%) had both HHV-6 and EBV DNA, 25 (34%) had only HHV-6, and 8 (11%) had only EBV. In contrast, of 10 cases of the mixed cellularity form of HD, 4 (40%) had both viruses, 1 had HHV-6 only, 4 had EBV only, and 1 had neither. HHV-6^+^/EBV^−^ patients were younger than the EBV^+^/HHV-6^−^ patients and 92% of the HHV-6^+^ lymph nodes contained variant B. However, Luppi et al.^22^ examined a large series of patients with HD in which HHV-6 DNA was found by both PCR and Southern blot analysis, did not detect either latent or lytic HHV-6 antigens in neoplastic cells, and detected only limited expression in Reed–Sternberg cells. Thus, the role of HHV-6 in any form of HD remains unclear. Recently, Lacroix et al.^23^ showed the transforming, transactivating and oncogenic properties of HHV-6B and localized the transforming activity into DR7 gene. Cells expressing viral DR7 protein revealed tumorigenic properties when injected into nude mice. The expression of DR7B protein in Reed-Sternberg cells from HD patients causes molecular alterations into the cells typical of the lymphoproliferative disorder. In particular, the oncoprotein protects infected cells from apoptosis by retaining human p53 within the cytoplasm and by increasing NF-kB cellular transcription factor. The action on NF-kB is mainly exerted through two mechanisms: the transactivation of the expression of its subunities p65 and p50–p105 and the direct interaction of DR7B with the assembled protein. Lastly, DR7B promotes the overexpression of Id2, inhibitor of E2A transcription factor, that negatively regulates cell differentiation.^23^ Further studies are needed to confirm a plausible pathogenetic role of HHV-6 infection in HD.

#### Non-Hodgkin’s lymphomas

Luppi et al.^24^ reported a higher frequency of HHV-6 DNA in a well-characterized series of patients with angioimmunoblastic T-cell lymphoma (AITL), a subtype of T-cell non-Hodgkin’s lymphoma (NHL), compared with other lymphoma subtypes and controls. These findings have been confirmed by Zhou et al.^25^ showing a clear association between histological progression of AITL and the detectable copy number of both EBV and HHV-6B in the AITL lesional tissue. While this increased viral load could reflect a role for HHV-6 in the pathogenesis and progression of AITL, it could also be the consequence of increasing dysfunction of the immune system during lymphoma progression. Immunohistochemical studies have so far failed to demonstrate HHV-6 antigens in the CD4+ T cells (the likely proliferating elements) within AITL lesions.

#### Leukemias

Persistent IL-2-regulated HHV-6 infection of adult T-cell leukemia cells causes T-cell leukemia to progress more rapidly,^26^ but in vivo studies have not yet confirmed a pathogenetic role for HHV-6 in this disease. Few other studies aiming to investigate the association of HHV-6 with acute leukemia have been reported. The largest study showed significant higher titres of HHV-6 antibodies in patients with acute myeloid leukemia, but not with acute lymphoblastic leukemia.^27^ Salonen et al.^28^ found that 40% of children with leukemia had IgM antibodies to HHV-6 compared to 7.7% of age- and sex-matched children with various neurological diseases. However, molecular studies have so far failed to show a higher rate of HHV-6 DNA in peripheral blasts from children with acute lymphoblastic leukemia compared with healthy subjects.^29^ A recent report found higher rates of seropositivity to human cytomegalovirus (HCMV) among patients with B-cell chronic lymphocytic leukemia than among healthy control subjects, although restricted only to some geographical areas, but the same was not true for seropositivity to HHV-6 (or EBV and HHV-7).^30^ In conclusion, with the possible exception of adult T-cell leukemia, available data do not lend support to a role for HHV-6 in human acute leukemias.

#### Non-malignant lymphatic tissue proliferation

Of interest, HHV-6 late antigens have so far been detected only in non-malignant lymph node proliferations, namely in cases of reactive lymphadenitis,^31^, ^32^ in which HHV-6 antigens appear to be restricted to CD4+T cells. HHV-6 late antigens have also been identified in cases of Rosai Dorfman disease, otherwise known as sinus histiocytosis with massive lymphadenopathy, a benign chronic disease, mainly affecting children and young adults and with no progression to lymphoma. HHV-6 infection appears to be restricted to follicular dendritic cells and, more significantly, to the abnormal histiocytes that represent the proliferating elements and the hallmark of this disease.^33^ (Table 1)

### HCMV

#### Epidemiology and biology

HCMV was simultaneously isolated from salivary glands by Rowe and Smith in 1956. This virus was designated ‘cytomegalovirus’ and the associated clinical syndrome was referred to as ‘cytomegalovirus inclusion disease’ because viral cytopathic effects typically result in cell swelling and intranuclear inclusions.

HCMV infection is widespread in the entire human population, with prevalence increasing with age. In Western Countries, seropositivity rates range 40–70%, while in developing countries are much higher.^34^ Using PCR, CMV viremia has been detected in about 98% of healthy individuals over 50 years of age. HCMV can be transmitted orally, sexually, and parenterally; primary infection may be subclinical in healthy subjects. Even asymptomatic carriers may at times shed HCMV in urine and saliva.^35^–^36^

HCMV productive infection (lytic cycle) is restricted to endothelial cells and fibroblasts, typically causing cell death and tissue damage in lung, liver, colon, brain and retina. Similarly to other herpesviruses, HCMV can establish lifelong latent infection in the host, mainly in macrophages and hematopoietic stem cells/progenitors (then passively transmitted to the mature myeloid progenies), and is a recognized cause of mononucleosis-like syndromes.^36^ (Table 1)

#### Large granular lymphocyte proliferation

In contrast to HHV-6, HCMV has not been proven to be involved in human cancer. However, Rodriguez-Caballero and colleagues^37^ suggested a role of HCMV in the pathogenesis of a specific subtype of large granular lymphocyte (LGL) proliferation involving CD4^+/^CD8^+/−dim^ T cells. In particular, they used microarray gene expression profile (GEP) to show that CD4^+^ T cells in patients with CD4^+^ LGL expansions, differ significantly from HCMV-specific memory CD4^+^ lymphocytes derived from healthy control individuals. The chronic antigenic stimulation of T cells by HCMV can lead to persistent monoclonal expansion of vβ13.1/CD4^+^ NKa^+^ CD8dim^+^ lymphocytes presenting a deregulation of genes involved in cell cycle progression, resistance to apoptosis and genetic instability. The observed deregulation of key genes allows these cells to accumulate in excess to what is required to control HCMV infection and to abnormally proliferate. (Table 1)

## γ HERPESVIRUSES

### HHV-8

#### Epidemiology and biology

Human herpesvirus-8 (HHV-8) was identified by Moore & Chang in 1994, from the Kaposi’s sarcoma (KS) tissues of patients with AIDS. HHV-8 is not ubiquitous in the general population: the infectious rates are low in the United Kingdom, United States and Asia, intermediate in Mediterranean countries and high in Central Africa. The seroprevalence of HHV-8 among blood donors ranges from 0.2% in Japan, to up to 10% in the United States, and to more than 50% in Africa,^38^ with rates in Italy and other Mediterranean countries falling between these percentages.^39^,^40^ HHV-8 is mainly spread by sexual route in non-endemic areas, while non-sexual transmission may be important in endemic areas where infection is usually acquired early in the childhood.

HHV-8 is classified as a γ-herpesvirus, related to EBV and Herpesvirus Saimiri. Like other herpesviruses, HHV-8 is a large, double-stranded DNA virus that replicates in the nucleus as a closer circular episome during latency, but linearizes during virion packaging and replication. The HHV-8 genome typically contains genes that are homologous to cellular genes involved in the control of cell cycle and apoptosis. Similarly to other herpesviruses, HHV-8 has evolved to persist within the lymphoid system and has shown an oncogenic potential. HHV-8 infection has been described in association with rare lymphoproliferative disorders, including primary effusion lymphoma (PEL), multicentric Castleman’s disease (MCD), and MCD-associated plasmablastic lymphoma, often occurring in AIDS patients. A subset of viral proteins is expressed in HHV-8-associated lymphoproliferative disorders and are involved in the viral lymphomagenesis. The viral proteins expressed in most PEL cells are the following: latency-associated nuclear antigen 1 (LANA -1), v-Cyclin, v-FLICE inhibitory protein (v-FLIP), v**-**interferon regulatory factor/LANA-2, Kaposin, v-Interleukein-6 (v-IL-6). LANA-1, v-Cyclin, v-FLIP and v-IL-6 are also expressed in most of the MCD cases. Two additional proteins, namely the K1 and the v-G-protein-coupled receptor (v-GPCR) are expressed in few cases of PEL and MCD.^41^ (Table 1)

#### Primary Effusion Lymphoma

PEL has been included in the WHO classification as a distinct entity among AIDS-associated NHLs, representing about 3**–**4% of all AIDS-NHLs.^42^–^44^ The lymphoma grows predominantly in serous effusions, without solid tumor masses in the affected body cavity,^45^–^47^ while involvement of lymph nodes,^48^ bone marrow^49^,^50^ or other tissues^51^ is occasionally seen. A number of continuous cell lines has been established from such lymphomatous effusions and peripheral blood of PEL patients.^52^ The PEL cells can include features of large cell immunoblastic and anaplastic lymphoma,^53^ and also sometimes express a more plasmacytoid cytology.^42^ PEL cells generally lack immunophenotypical expression of differentiated B- or T- cell antigens, but for MUM1 and CD138, reflecting their post-germinal centre B-cell origin. Consistent with this, the gene expression profile analysis suggests a plasmablastic derivation of PEL cells.^54^,^55^ They express cell activation associated markers, including HLA-DR, CD23, CD25, CD30 and CD38, and the epithelial membrane antigen whereas adhesion markers are variably expressed.^43^,^52^ The B cell lineage derivation of PEL cells is established on the basis of clonal rearrangements of the heavy immunoglobulin (Ig) genes,^45^,^52^ and the PCR-based findings of a preferential expression of certain lambda light chain genes in AIDS-related PELs, suggesting clonal proliferation by an antigen selection process.^56^ A few cases of AIDS-related PEL did not demonstrate Ig gene rearrangements, consistent with a polyclonal pattern of lymphoproliferation.^47^ In contrast to other non Hodgkin’s B-cell lymphoma types, neither c-MYC nor other proto-oncogene rearrangements were detected in PELs.^54^ Likewise, a wild type of the tumor suppressor p53 gene is expressed by PELs, while mutations of the BCL-6 5′ non-coding regions have been documented in most of the cases.^52^ PELs show complex karyotypes, the most frequent chromosomal abnormalities being trisomy 7, 12 and aberrations of chromosomal bands 1q21-q25.^52^ Virtually all reported cases of PEL have a relatively high number of HHV-8 DNA copies (40–150) per cell, most cells being latently infected and relatively few permissive for lytic infection as obtained in cultured PEL lines. Analysis of HHV-8 terminal repeats (TR) by pulsed-field gel electrophoresis has shown monoclonal or oligoclonal fused TR fragments in all examined cases of PEL, suggesting HHV-8 infection of clonogenic cells, supporting an etiologic role of the virus in these lymphoproliferations.^57^ EBV co-infection is detected in many cases of PEL, also with a monoclonal infection pattern and with a restricted antigen expression pattern of latency. Human interleukin-6 (IL-6) and -10 (IL-10), v-IL-6 and vascular endothelial growth factor (VEGF) are the major growth factors released and used by PEL cells for autocrine growth stimulation.^52^,^58^–^60^ The occurrence of PEL in a non-AIDS setting appears to be very rare and has been reported in very few cases of solid organ transplant patients,^50^,^61^,^62^ and a few cases have also been described in HIV negative elderly men, most of them originating from HHV-8 endemic areas.^63^,^64^ The clinical outcome of AIDS-related and post-transplant PEL is very poor, with a median survival from 2 to 6 months, despite chemotherapy.^50^,^52^,^57^ Decreasing CD4+ cell counts seem to be the most important indicator of progression of AIDS-related PEL.^65^ In HIV negative patients, PEL may have a more indolent clinical course without specific therapy and may initially respond to drainage procedures.^63^ Recently, it has been reported that azidothymidine and interferon-α induce apoptosis in PEL cells either in vitro or in vivo,^66^,^67^ and PEL remission was observed in a patient on anti-retroviral therapy.^68^ We have demonstrated that cidofovir at high doses induces in vitro apoptosis in PEL cell lines and PEL remission in four HIV negative, elderly Italian men treated with intrapleural/intraperitoneal injections of cidofovir, who had recurrent effusions not responding either to pleural/peritoneal drainages or to chemotherapy.^69^,^70^ Recent in vitro data have shown that glycyrrhizic acid, contained in the licorice root, induces apoptosis of PEL cells, by down-regulating the synthesis of the HHV-8 LANA-1.^71^ Other approaches have recently been considered for the treatment of PEL, based on the targeting of viral gene products,^72^,^73^ providing the basis for new therapeutic options for PEL patients who are generally poor candidates for aggressive chemotherapy.

#### Multicentric Castleman’s Disease (MCD) and MCD-associated plasmablastic lymphoma

MCD of plasma cell type is an atypical lymphoproliferative disorder, which is histologically characterized by abundance and prominent alterations of the germinal centers, marked plasmacytic infiltration, and vascular hyperplasia.^74^ Two types of malignancies, lymphoma and KS, have been reported to occur during the course of MCD in 18% and 13% of cases respectively.^74^ HHV-8 DNA sequences have been detected in most of MCD cases occurring in HIV positive patients, but only in few HIV negative cases.^75^–^78^ HHV-8 infection is also found in most MCD patients with associated POEMS (polyneuropathy, organomegaly, endocrinopathy, M protein, skin changes) syndrome.^79^ One case of HHV-8 positive MCD has also been reported in a renal transplant patient with KS.^80^ HHV-8 positive MCD cells, expressing LANA-1, morphologically resemble plasmablasts and are localized in the mantle zone of the follicles.^81^ These plasmablasts show λ light-chain restriction and coalesce to form microscopic lymphomas in some MCD cases, which could herald the development of frank HHV-8 positive plasmablastic lymphoma.^82^–^84^ A role in the pathogenesis of MCD for an over-expression of IL-6, a cytokine which promotes B cell survival and proliferation, has been proposed.^74^ The expression of v-IL-6 in a proportion of HHV-8 infected MCD cells^85^–^88^ thus appears to support such a pathogenic mechanism. This is consistent with findings that exacerbations of systemic symptoms in MCD correlate with an increase in HHV-8 viral load together with IL-6 and IL-10, which thus represent markers of disease activity.^89^,^90^ Recent studies suggest that HHV-8 positive MCD cases have a more aggressive clinical course and a poorer prognosis.^82^ With regard to therapy, single agent chemotherapy with vinblastine is the most effective therapeutic option and may prolong survival.^91^ A patient with MCD has successfully been treated with retinoic acid and prednisone.^92^ Ganciclovir has also been effective in attenuating the constitutional symptoms in some cases of HIV-associated HHV-8 positive MCD cases.^93^ Recently, treatment of MCD with humanized anti-IL-6 receptor antibody has been reported to be safe and to alleviate chronic inflammatory symptoms and wasting in a series of 28 patients, followed-up for 60 weeks.^94^

#### Other diseases

The pathogenetic association between HHV-8 infection and the development of multiple myeloma, proposed by Rettig and colleagues,^95^ has not been confirmed.^96^–^98^ HHV-8 infection is certainly rare in lymphoproliferative diseases other than PEL or MCD, both in HIV positive and HIV negative subjects. Moreover, the occurrence of HHV-8 positive solid lymphomas, usually extranodal and extracavitary, but with pathobiological features mimicking those of PEL, has been described in AIDS as well as in HIV negative patients.^99^ HHV-8 infection was documented in association with hepatitis C virus infection in one case of plasma cell leukemia,^100^ and three HIV negative cases of a germinotropic lymphoproliferative disorder characterized by plasmablasts coinfected by HHV-8 and EBV have also been described.^101^ HHV-8 DNA and LANA-1 antigens have been detected in liver, lung and bone marrow tissues from patients affected with common variable immunodeficiency and granulomatous/lymphocytic interstitial lung disease, suggesting a pathogenetic viral role in this disorder.^102^ HHV-8 DNA was also found in a single case of primary cerebral lymphoma, in a woman who had received long-term steroid therapy for uveitis, suggesting that HHV-8 infection may be occasionally involved in a lymphoproliferation process associated with iatrogenic immunesuppression.^103^ Consistent with this, the occurrence of an EBV negative, HHV-8 positive, monoclonal, lymphoproliferative disease of polymorphic type has recently been reported in a HHV-8 seronegative Jewish man, nine months after receiving a kidney from his HHV-8 seropositive father.^104^ Non malignant plasmacytic proliferations have also been reported in two solid organ transplant patients^105^ as well as in a few Italian cases of HIV negative angioimmunoblastic lymphadenopathy.^106^ Interestingly, a few cases of benign lymphadenopathy with germinal center hyperplasia and increased vascularity in which HHV-8 DNA sequences were detected, have been reported in HIV negative^75^,^106^ and HIV positive^106^,^107^ young adults. The only one case of well documented HHV-8 primary infection in HIV positive subjects has been reported to be associated with the development of fever, splenomegaly and a cervical lymphadenopathy, characterized by angiolymphoid hyperplasia.^108^ Thus, the above mentioned histologic features of florid follicular hyperplasia and increased vascularity, which are observed also in MCD, are likely to represent the distinct histologic pattern of lymphoid response induced by HHV-8. Interestingly, a lymphoproliferative disease characterized by persistent angiofollicular lymphadenopathy is induced in simian immunodeficiency-virus infected Rhesus Macaques, following infection with the simian homologue of HHV-8.^109^

It is also likely that, as with other human herpesviruses, a HHV-8 primary infection or reactivation, is manifested by non neoplastic pathological changes. Thus, HHV-8 DNA has been detected in the pathologic lung tissue of HIV negative and positive patients with interstitial pneumonitis.^110^,^111^ Fever, cutaneous rash and hepatitis have also been reported in an Italian patient with NHL, who received autologous peripheral blood stem cell (PBSC) transplantation and showed HHV-8 reactivation.^112^ Recently, we had also the possibility to study primary HHV-8 infection in two patients four months after kidney transplantation from the same HHV-8-seropositive cadaveric donor. Seroconversion and viremia coincided with development of a disseminated KS in one patient and with an acute syndrome of fever, splenomegaly, cytopenia, and marrow failure with plasmacytosis in the other patient.^113^ We also reported a further case of HHV-8 reactivation associated with fever and marrow aplasia with plasmacytosis in a patient with NHL, after autologous PBSC transplantation. HHV-8 transcripts and latency associated nuclear antigen were expressed in the immature myeloid progenitors of the aplastic marrow of these patients.^113^ In recent studies we and others have observed that HHV-8 may also exert a myelosuppressive effect in vitro,^114^,^115^ suggesting that HHV-8 could also be implicated in the complex pathophysiology of cytopenias often occurring in HIV infected patients.^116^ (Table 1)

## Figures and Tables

**Table 1:** Main biological, epidemiological and hematologic features of human β-herpesviruses and HHV-8 infections.

Herpes Virus	Tropism	Epidemiology	Non-neoplastic hematologic manifestations	Associations with neoplastic diseases
HHV-6	Peripheral blood mononuclear cells (mainly T lymphocytes and monocytes); hematopoietic progenitor cells; neuroglial cells; salivary gland epithelial cells. Lifelong persistence also as chromosomal genome integration.	Worldwide diffusion (variants A and B, with 90% nucleotide identity), infection early in life. Transmission: saliva, in utero, blood, organ transplant.	Mononucleosis-like syndrome, reactive lymphadenopathies, hemophagocytic syndrome, pancytopenias. Possible role in Rosai Dorfman Disease.	Possible role in Angioimmunoblastic T-cell Lymphoma and in Hodgkin’s disease.
HCMV	Hematopoietic progenitor cells (mainly monocyte-macrophages); peripheral blood mononuclear cells; endothelial cells and fibroblasts.	Worldwide diffusion, seropositivity increasing with age (98% over 50 years old). Transmission: saliva, in utero, sexual route, blood, organ transplant.	Mononucleosis-like syndrome, hemophagocytic syndrome, pancytopenias.	Possible role in Large Granular Lymphocyte lymphocytosis.
HHV-8	B-lymphocytes; hematopoietic progenitors cells; microvascular endothelial cells (lymphatic and blood vascular cells).	Not ubiquitary diffusion: seroprevalence 30–70% in endemic African areas, 10–25% in Mediterranean areas. Transmission: vertical in endemic areas, sexual in non-endemic areas; organ transplant, blood?	Reactive lymphadenopathies, hemophagocytic syndrome, pancytopenias, bone marrow aplasia in organ transplant patients.	Kaposi’s Sarcoma, Primary Effusion Lymphoma, Multicentric Castleman’s Disease/plasmablastic lymphoma.

## References

[1] Comoli P, Locatelli F (2009). T-cell therapy for the treatment of Epstein Barr virus-associated malignancies. Immunotheapy Insights.

[2] Caserta MT, Mock DJ, Dewhurst S (2001). Human Herpes Virus 6. Clin Inf Dis.

[3] Clark DA (2000). Human herpes virus 6. Rev Med Virol.

[4] Santoro F, Kennedy PE, Locatelli G, Malnati MS, Berger EA, Lusso P (1999). CD46 is a cellular receptor for human herpesvirus 6. Cell.

[5] Ward KN, Leong HN, Nacheva EP, Howard J, Atkinson CE, Davies NW, Griffiths PD, Clark DA (2006). Human herpesvirus 6 chromosomal integration in immunocompetent patients results in high levels of viral DNA in blood, sera, and hair follicles. J Clin Microbiol.

[6] Potenza L, Barozzi P, Masetti M, Pecorari M, Bresciani P, Gautheret-Dejean A, Riva G, Vallerini D, Tagliazucchi S, Codeluppi M, Di Benedetto F, Gerunda GE, Narni F, Torelli G, Luppi M (2009). Prevalence of human herpesvirus-6 chromosomal integration (CIHHV-6) in Italian solid organ and allogeneic stem cell transplant patients. Am J Transplant.

[7] Torelli G, Barozzi P, Marasca R, Cocconcelli P, Merelli E, Ceccherini-Nelli L, Ferrari S, Luppi M (1995). Targeted integration of human herpesvirus 6 in the p arm of chromosome 17 of human peripheral blood mononuclear cells in vivo. J Med Virol.

[8] Morris C, Luppi M, McDonald M, Barozzi P, Torelli G (1999). Fine mapping of an apparently targeted latent human herpesvirus type 6 integration site in chromosome band 17p13.3. J Med Virol.

[9] Daibata Taguchi T, Taguchi H, Miyoshi I (1998). Integration of human herpesvirus 6 in a Burkitt’s lymphoma cell line. Br J Haematol.

[10] Daibata M, Taguchi T, Nemoto Y, Taguchi H, Miyoshi I (1999). Inheritance of chromosomally integrated human herpesvirus 6 DNA. Blood.

[11] Puri RK, Leland P, Razzaque A (199). Antigen(s)-specific tumour infiltrating lymphocytes from tumour induced by fhuman herpesvirus-6 (HHV-6) DNA transfected NIH 3T3 transformants. Clin Exp Immunol.

[12] Ogata M, Satou T, Kawano R, Takakura S, Goto K, Ikewaki J, Kohno K, Ikebe T, Ando T, Miyazaki Y, Ohtsuka E, Saburi Y, Saikawa T, Kadota J (2009). Correlations of HHV-6 viral load and plasma IL-6 concentration with HHV-6 encephalitis in allogeneic stem cell transplant recipients. Bone Marrow Transplant.

[13] Razzaque A (1990). Oncogenic potential of human herpesvirus-6 DNA. Oncogene.

[14] Razzaque A, Williams O, Wang J, Rhim JS (1993). Neoplastic transformation of immortalized human epidermal keratinocytes by two HHV-6 DNA clones. Virology.

[15] Kashanchi F, Araujo J, Doniger J, Muralidhar S, Hoch R, Khleif S, Mendelson E, Thompson J, Azumi N, Brady JN, Luppi M, Torelli G, Rosenthal LJ (1997). Human herpesvirus 6 (HHV-6) ORF-1 transactivating gene exhibits malignant transforming activity and its protein binds to p53. Oncogene.

[16] Doniger J, Muralidhar S, Rosenthal LJ (1999). Human cytomegalovirus and human herpesvirus 6 genes that transform and transactivate. Clin Microbiol Rev.

[17] Takemoto M, Shimamoto T, Isegawa Y, Yamanishi K (2001). The R3 region, one of three major repetitive regions of human herpesvirus 6, is a strong enhancer of immediate-early gene U95. J Virol.

[18] Campbell KJ, Perkins ND (2006). Regulation of NF-kappaB function. Biochem Soc Symp.

[19] Torelli G, Marasca R, Luppi M, Selleri L, Ferrari S, Narni F, Mariano MT, Federico M, Ceccherini-Nelli L, Bendinelli M, Montagnani G, Montorsi M, Artusi T (1991). Human herpesvirus-6 in human lymphomas: Identification of specific sequences in Hodgkin’s lymphomas by polymerase chain reaction. Blood.

[20] Krueger GR, Huetter ML, Rojo J, Romero M, Cruz-Ortiz H (2001). Human herpesviruses HHV-4 (EBV) and HHV-6 in Hodgkin’s and Kikuchi’s diseases and their relation to proliferation and apoptosis. Anticancer Res.

[21] Lacroix A, Jaccard A, Rouzioux C, Piguet C, Petit B, Bordessoule D, Ranger-Rogez S (2007). HHV-6 and EBV DNA quantitation in lymph nodes of 86 patients with Hodgkin’s lymphoma. J Med Virol.

[22] Luppi M, Barozzi P, Garber R, Maiorana A, Bonacorsi G, Artusi T, Trovato R, Marasca R, Torelli G (1998). Expression of human herpesvirus-6 antigens in benign and malignant lymphoproliferative diseases. Am J Pathol.

[23] Lacroix A, Collot-teixeira S, Mardivirin L, Jaccard A, Petit B, Piguet C, Sturtz F, Preux PM, Bordeussoule D, Ranger-Rogez S (2010). Involvement of Human Herpesvirus-6 Variant B in classic Hodgking’s Lymphoma via DR7 oncoprotein. Clin Cancer Res.

[24] Luppi M, Marasca R, Barozzi P, Artusi T, Torelli G (1993). Frequent detection of human herpesvirus-6 sequences by polymerase chain reaction in paraffin-embedded lymph nodes from patients with angioimmunoblastic lymphadenopathy and angioimmunoblastic lymphadenopathy-like lymphoma. Leuk Res.

[25] Zhou Y, Attygalle AD, Chuang SS, Diss T, Ye H, Liu H, Hamoudi RA, Munson P, Bacon CM, Dogan A, Du MQ (2007). Angioimmunoblastic T-cell lymphoma: Histological progression associates with EBV and HHV6B viral load. Br J Haematol.

[26] Ojima T, Abe K, Ohyashiki JH, Shirakata M, Yamamoto K (2005). IL-2-regulated persistent human herpesvirus-6 B infection facilitates growth of adult T cell leukemia cells. J Med Dent Sci.

[27] Levine PH, Ablashi DV, Saxinger WC, Connelly RR (1992a). Antibodies to human herpes virus-6 in patients with acute lymphocytic leukemia. Leukemia.

[28] Salonen MJ, Siimes MA, Salonen EM, Vaheri A, Koskiniemi M (2002). Antibody status to HHV-6 in children with leukaemia. Leukemia.

[29] Barozzi P, Luppi M, Marasca R, Trovato R, Ceccherini-Nelli L, Torelli G (1995). Human herpesvirus-6 genome in acute lymphoblastic leukemia: Evidence against an etiologic relationship. Acta Haematol.

[30] Steininger C, Rassenti LZ, Vanura K, Eigenberger K, Jager U, Kipps TJ, Mannhalter C, Stilgenbauer S, Popow-Kraupp T (2009). Relative seroprevalence of human herpes viruses in patients with chronic lymphocytic leukaemia. Eur J Clin Invest.

[31] Akashi K, Eizuru Y, Sumiyoshi Y, Minematsu T, Hara S, Harada M, Kikuchi M, Niho Y, Minamishima Y (1993). Brief report: Severe infectious mononucleosis-like syndrome and primary human herpesvirus 6 infection in an adult. N Engl J Med.

[32] Maric I, Bryant R, Abu-Asab M, Cohen JI, Vivero A, Jaffe ES, Raffeld M, Tsokos M, Banks PM, Pittaluga S (2004). Human herpesvirus-6- associated acute lymphadenitis in immunocompetent adults. Mod Pathol.

[33] Levine PH, Jahan N, Murari P, Manak M, Jaffe ES (1992). Detection of human herpesvirus 6 in tissues involved by sinus histiocytosis with massive lymphadenopathy (Rosai–Dorfman disease). J Infect Dis.

[34] Blut Arbeitskreis (2010). Untergruppe “Bewertung Blutassoziierter Krankheitserreger”. Human Cytomegalovirus (HCMV). Transfus Med Hemother.

[35] Boeckh M, Geballe AP (2011). Cytomegalovirus: pathogen, paradigm, and puzzle. J Clin Invest.

[36] Sinzger C, Digel M, Jahn G (2008). Cytomegalovirus cell tropism. Curr Top Microbiol Immunol.

[37] Rodríguez-Caballero A, García-Montero AC, Bárcena P, Almeida J, Ruiz-Cabello F, Tabernero MD, Garrido P, Munoz-Criado S, Sandberg Y, Langerak AW, González M, Balanzategui A, Orfao A (2008). Expanded cells in monoclonal TCR alphabeta+/ CD4+/ NKa+/ CD8-/+dim T-LGL lymphocytosis recognize hCMV antigens. Blood.

[38] Lennette ET, Blackbourn DJ, Levy JA (1996). Antibodies to human herpesvirus type 8 in the general population and in Kaposi’s sarcoma patients. Lancet.

[39] Whitby D, Luppi M, Barozzi P, Boshoff C, Weiss RA, Torelli G (1998). Human herpesvirus 8 seroprevalence in blood donors and lymphoma patients from different regions of Italy. J Natl Cancer Inst.

[40] Simpson GR, Schulz TF, Whitby D (1996). Prevalence of Kaposi’s sarcoma associated herpesvirus infection measured by antibodies to recombinant capsid protein and latent immunofluorescence antigen. Lancet.

[41] Damania B (2004). Oncogenic γ-herpesviruses: comparison of viral proteins involved in tumorigenesis. Nat Rev Microbiol.

[42] Carbone A, Gaidano G (1997). HHV-8 positive body-cavity-based lymphoma: a novel lymphoma entity. Br J Haematol.

[43] Harris NL, Jaffe ES, Diebold J, Flandrin G, Muller-Hermelink K, Vardiman J, Lister TA, Bloomfield CD, World Health Organization (1999). Classification of neoplastic diseases of the hematopoietic and lymphoid tissues: report of the clinical advisory committee meeting-Airlie House, Virginia, November 1997. J Clin Oncol.

[44] Simonelli C, Spina M, Cinelli R, Talamini R, Tedeschi R, Gloghini A, Vaccher E, Carbone A, Tirelli U (2003). Clinical features and outcome of primary effusion lymphoma in HIV-infected patients: a single-institution study. J Clin Oncol.

[45] Cesarman E, Chang Y, Moore PS, Said JW, Knowles DM (1995). Kaposi’ sarcoma-associated herpesvirus-like DNA sequences in AIDS-related body-cavity-based lymphomas. New Engl J Med.

[46] Nador RG, Cesarman E, Chadburn A, Dawson DB, Ansari MQ, Said J, Knowles DM (1996). Primary effusion lymphoma: a distinct clinico-pathologic entity associated with the Kaposi’s sarcoma-associated herpesvirus. Blood.

[47] Komanduri KVJ, Luce JA, McGrath MS, Herndier BG, Ng VL (1996). The natural history and molecular heterogeneity of HIV-associated primary malignant lymphomatous effusions. J Acquir Immun Defic.

[48] Ariad S, Benharroch D, Lupu L, Davidovici B, Dupin N, Boshoff C (2000). Early peripheral lymph node involvement of human herpesvirus 8-associated body cavity-based lymphoma in a human immunodeficiency virus-negative patient. Arch Pathol Lab Med.

[49] Boshoff C, Gao S-J, Healy LE, Matthews S, Thomas AJ, Coignet L, Warnke RA, Strauchen JA, Matutes E, Kamel OW, Moore PS, Weiss RA, Chang Y (1998). Establishing a KSHV+ cell line (BCP-1) from peripheral blood and characterizing its growth in Nod/SCID mice. Blood.

[50] Dotti G, Fiocchi R, Motta T, Facchinetti B, Chiodini B, Boleri GM, Gavazzeni G, Barbui T, Rambaldi S (1999). Primary effusion lymphoma after heart transplantation: a new entity associated with human herpesvirus-8. Leukemia.

[51] DePond W, Said JW, Tasaka T, de Vos S, Kahn D, Cesarman E, Knowles DM, Koeffler HP (1997). Kaposi’s sarcoma-associated herpesvirus and human herpesvirus 8 (KSHV/HHV-8)-associated lymphoma of the bowel. Am J Surg Pathol.

[52] Drexler HG, Huphoff CC, Gaidano G, Carbone A (1998). Lymphoma cell lines: in vitro models for the study of HHV-8 + primary effusion lymphomas (body-cavity based lymphomas). Leukemia.

[53] Jaffe E (1996). Primary body cavity-based AIDS-related lymphomas. Evolution of a new disease entity. Am J Clin Pathol.

[54] Carbone A (2003). Emerging pathways in the development of AIDS-related lymphomas. The Lancet Oncology.

[55] Klein U, Gloghini A, Gaidano G, Chadburn A, Cesarman E, Dalla-Favera R, Carbone A (2003). Gene expression profile analysis of AIDS-related primary effusion lymphoma (PEL) suggests a plasmablastic derivation and identifies PEL-specific transcripts. Blood.

[56] Fais F, Gaidano G, Capello D, Gloghini A, Ghiotto F, Roncella S, Carbone A, Chiorazzi N, Ferrarini M (1999). Immunoglobulin V region gene use and structure suggest antigen selection in AIDS-related primary effusion lymphomas. Leukemia.

[57] Judde J-G, Lacoste V, Brière J, Kassa-Kelembho E, Clyti E, Couppiè P, Buchrieser C, Tulliez M, Morvan J, Gessain A (2000). Monoclonality or oligoclonality of human herpesvirus 8 terminal repeat sequences in Kaposi’s sarcoma and other diseases. J Natl Canc Inst.

[58] Jones KD, Aoki Y, Chang Y, Moore PS, Yarchoan R, Tosato G (1999). Involvement of interleukin-10 (IL-10) and viral IL-6 in the spontaneous growth of Kaposi’s sarcoma herpesvirus-associated infected primary effusion lymphoma cells. Blood.

[59] Aoki Y, Jaffe ES, Chang Y, Jones K, Teruya-Feldstein J, Moore PS, Tosato G (1999). Angiogenesis and hematopoiesis induced by Kaposi’s sarcoma-associated herpesvirus-encoded interleukin-6. Blood.

[60] Aoki Y, Tosato G (1999). Role of vascular endothelial growth factor/vascular permeability factor in the pathogenesis of Kaposi sarcoma-associated herpesvirus-infected primary effusion lymphomas. Blood.

[61] Jones D, Ballestas ME, Kaye KM, Gulizia JM, Winters GL, Fletcher J, Scadden DT, Aster JC (1998). Primary effusion lymphoma and Kaposi’s sarcoma in a cardiac-transplant recipient. N Engl J Med.

[62] Shaw RN, Waller EK, Offermann MK (2002). Induction of human herpesvirus 8 gene expression in a post-transplantation primary effusion lymphoma cell line. Leuk Lymphoma.

[63] Strauchen JA, Hauser D, Burstein D, Jimenez R, Moore PS, Chang Y (1996). Body-cavity-based malignant lymphoma containing Kaposi sarcoma-associated herpesvirus in an HIV-negative man with previous Kaposi sarcoma. Ann Intern Med.

[64] Ascoli V, Lo Coco F, Torelli G, Vallisa D, Cavanna L, Bergonzi C, Luppi M (2002). Human herpesvirus 8-associated primayi effusion lymphoma in HIV-patients: a clinico epidemiologic variant resembling classic Kaposi’s sarcoma. Haematologica.

[65] Simonelli C, Tedeschi R, Gloghini A, Bortolin MT, Spina M, Bidoli E, Cinelli R, De Paoli P, Carbone A, Tirelli U (2005). Characterization of immunologic and virological parameters in HIV-infected patients with primary effusion lymphoma during antiblastic therapy and highly active antiretroviral therapy. Clin Infect Dis.

[66] Lee RK, Cai J-P, Deyev V, Gill PS, Cabral L, Wood C, Agarwal RP, Xia W, Boise LH, Podack E, Harrington WJ (1999). Azidothymidine and interferon-α induce apoptosis in herpesvirus-associated lymphomas. Cancer Res.

[67] Ghosh SK, Wood C, Boise LH, Mian AM, Deyev VV, Feuer G, Toomey NL, Shank NC, Cabral L, Barber GN, Harrington WJ (2003). Potentiation of TRAIL-induced apoptosis in primary effusion lymphoma through azidothymidine-mediated inhibition of NF-kappa B. Blood.

[68] Oksenhendler E, Clauvel JB, Jouveshomme S, Davi F, Mansour G (1998). Complete remission of a primary effusion lymphoma with antiretroviral therapy. Am J Hematol.

[69] Luppi M, Trovato R, Barozzi P, Vallisa D, Rossi G, Re A, Ravazzini L, Potenza L, Riva G, Morselli M, Longo G, Cavanna L, Roncaglia R, Torelli G (2005). Treatment of herpesvirus associated primary effusion lymphoma with intracavity cidofovir. Leukemia.

[70] Halfdanarson TR, Markovic SN, Kalokhe U, Luppi M (2006). A non-chemotherapy treatment of a primary effusion lymphoma: durable remission after intracavitary cidofovir in HIV negative PEL refractory to chemotherapy. Ann Oncol.

[71] Curreli F, Friedman-Kien AE, Flore O (2005). Glycyrrhizic acid alters Kaposi sarcoma-associated herpesvirus latency, triggering p53-mediated apoptosis in transformed B lymphocytes. J Clin Invest.

[72] Cohen JI (2005). Licking latency with licorice. J Clin Invest.

[73] Klass CM, Offermann MK (2005). Targeting human herpesvirus-8 for the treatment of Kaposi’s sarcoma and primary effusion lymphoma. Curr Opin Oncol.

[74] Peterson BA, Frizzera G (1993). Multicentric Castleman’s disease. Semin Oncol.

[75] Soulier J, Grollet L, Oksenhendler E, Cacoub P, Cazals-Hatem D, Babinet P, d’Agay M-F, Clauvel J-P, Raphael M, Degos L, Sigaux F (1995). Kaposi’s sarcoma-associated herpesvirus-like DNA sequences in multicentric Castleman’s disease. Blood.

[76] Gessain A, Sudaka A, Brière J, Fouchard N, Nicola M-A, Rio B, Arborio M, Troussard X, Audouin J, Diebold J, de Thè G (1996). Blood.

[77] Corbellino M, Poirel L, Aubin JT, Paulli M, Magrini U, Bestetti G, Galli M, Parravicini C (1996). The role of human herpesvirus 8 and Epstein-Barr virus in the pathogenesis of giant lymph node hyperplasia (Castleman’s disease). Clin Infect Dis.

[78] Barozzi P, Luppi M, Masini L, Marasca R, Savarino M, Morselli M, Ferrari MG, Bevini M, Bonacorsi G, Torelli G (1996). Lymphotropic herpes virus (EBV, HHV-6, HHV-8) DNA sequences in HIV negative Castleman’s disease. J Clin Pathol Mol Pathol.

[79] Bèlec L, Mohamed AS, Authier F-J, Hallouin M-C, Soe AM, Cotigny S, Gaulard P, Gherardi RK (1999). Human herpesvirus 8 infection in patients with POEMS syndrome-associated multicentric Castleman’s disease. Blood.

[80] Parravicini C, Olsen SJ, Capra M, Poli F, Sirchia G, Gao SJ, Berti E, Nocera A, Rossi E, Bestetti G, Pizzuto M, Galli M, Moroni M, Moore PS, Corbellino M (1997). Risk of Kaposi’s sarcoma-associated herpesvirus transmission from donor allografts among Italian post-transplant Kaposi’s sarcoma patients. Blood.

[81] Dupin N, Fisher C, Kellam P, Ariad S, Tulliez M, Franck N, Van Marck E, Salmon D, Gorin I, Escande J-P, Weiss RA, Alitalo K, Boshoff C (1999). Distribution of human herpesvirus-8 latently infected cells in Kaposi’s sarcoma, multicentric Castleman’s disease, and primary effusion lymphoma. Proc Natl Acad Sci.

[82] Dupin N, Diss TL, Kellam P, Tulliez M, Du M-Q, Sicard D, Weiss RA, Isaacson PG, Boshoff C (2000). HHV-8 is associated with a plasmablastic variant of Castleman disease that is linked to HHV-8-positive plasmablastic lymphoma. Blood.

[83] Du MQ, Liu H, Diss TC, Ye H, Hamoudi RA, Dupin N, Meignin V, Oksehendler E, Boshoff C, Isaacson PG (2001). Kaposi sarcoma-associated herpesvirus infects monotypic (IgM lambda) but polyclonal naive B cells in Castleman disease and associated lymphoproliferative disorders. Blood.

[84] Oksenhendler E, Boulanger E, Galicier L, Du M-Q, Dupin N, Diss TC, Hamoudi R, Daniel M-T, Agbalika F, Boshoff C, Clauvel J-P, Isaacson PG, Meignin V (2002). High incidence of Kaposi sarcoma-associated herpesvirus-related non–Hodgkin lymphoma in patients with HIV infection and multicentric Castleman disease. Blood.

[85] Parravicini C, Corbellino M, Paulli M, Magrini U, Lazzarino M, Moore PS, Chang Y (1997). Expression of a virus-derived cytokine, KSHV vIL-6 in HIV sieronegative Castleman’s disease. Am J pathol.

[86] Parravicini C, Chandran B, Corbellino M, Berti E, Paulli M, Moore PS, Chang Y (2000). Differential viral protein expression in Kaposi’s sarcoma. Am J Pathol.

[87] Staskus KA, Sun R, Miller G, Racz P, Jaslowski A, Metroka C, Brett-Smith H, Haase AT (1999). Cellular tropism and viral interleukin-6 expression distinguish human herpesvirus 8 involvement in Kaposi’ sarcoma, primary effusion lymphoma, and Castleman’s disease. J Virol.

[88] Luppi M, Barozzi P, Maiorana A, Trovato R, Marasca R, Morselli M, Cagossi K, Torelli G (1999). Expression of cell-homologous genes of human herpesvirus-8 in human immunodeficiency virus-negative lymphoproliferative diseases. Blood.

[89] Grandadam MJ, Gorin I, Blum L, Kernbaum S, Sicard D, Buisson Y, Agut H, Escande JB, Huraux JM (1997). Exacerbations of clinical symptoms in human immunodeficiency virus type-1 infected patients with multicentric Castleman’s disease are associated with a high increase in Kaposi’s sarcoma herpesvirus DNA load in peripheral blood mononuclear cells. J Infect Dis.

[90] Oksenhendler E, Carcelain G, Aoki Y, Boulanger E, Maillard A, Clauvel J-P, Agbalika F (2000). High levels of human herpesvirus 8 viral load, human interleukin-6, interleukin-10, and C reactive protein correlate with exacerbation of multicentric Castleman disease in HIV-infected patients. Blood.

[91] Oksenhendler E, Cacoub P, Welker Y, Cadranel J, Cazals-Hatem D, Autran B, Clauvel JP, Raphael M (1996). Multicentric Castleman disease in HIV infection: a clinical and pathological study of 20 patients. AIDS.

[92] Rieu P, Droz D, Gessain A, Grunfeld JB, Hermine O (1999). Retinoic acid for treatment of multicentric Castleman disease. Lancet.

[93] Casper C, Nicholas G, Huang M-L, Corey L, Wald A (2004). Remission of HHV-8 and HIV-associated multicentric Castleman disease with ganciclovir treatment. Blood.

[94] Nishimoto N, Kanakura Y, Aozasa K, Johkoh T, Nakamura M, Nakano S, Nakano N, Ikeda Y, Sasaki T, Asaoku H, Kumagai S, Kodama F, Nakahara H, Hagihara K, Yoshizaki K, Kishimoto T (2005). Humanized anti-interleukin receptor antibody treatment of multicentric Castleman disease. Blood.

[95] Rettig MB, Ma HJ, Vescio RA, Pold M, Schiller G, Belson D, Savage A, Nishikubo C, Wu C, Fraser J, Said JW, Berenson JR (1997). Kaposi’s sarcoma-associated herpesvirus infection of bone marrow dendritic cells from multiple myeloma patients. Science.

[96] Yi O, Ekman M, Anton D, Bergenbrant S, Osterborg A, Goergii-Hemming P, Holm G, Nilsson K, Bibefeld P (1998). Blood dendritic cells from myeloma patients are not infected with Kaposi’s sarcoma-associated herpesvirus (KSHV/HHV-8). Blood.

[97] Tarte K, Chang Y, Klein B (1999). Kaposi’s sarcoma-associated herpesvirus and multiple myeloma: lack of criteria for causality. Blood.

[98] Ablashi DV, Chatlynne L, Thomas D, Bourboulia D, Rettig MB, Vescio RA, Viza D, Gill P, Kyle RA, Berenson JR, Whitman JE (2000). Lack of serologic association of human herpesvrus-8 (KSHV) in patients with monoclonal gammopathy of undetermined significance with and without progression to multiple myeloma. Blood.

[99] Carbone A, Gloghini A, Vaccher E, Cerri M, Gaidano G, Dalla-Favera R, Tirelli U (2005). Kaposi’s sarcoma-associated herpesvirus/human herspesvirus 8-positive solid lymphomas. A tissue-based variant of primary effusion lymphomas. J Mol Diagn.

[100] Hermouet S, Corre I, Gassin M, Bigot-Corbel E, Sutton CA, Casey JW (2003). Hepatitis C virus, human herpesvirus 8, and the development of plasma cell leukemia. New Engl J Med.

[101] Du MQ, Diss TC, Liu H, Ye H, Hamoudi RA, Cabecadas J, Dong HY, Harris NL, Chan JK, Rees JW, Dogan A, Isaacson PG (2002). KSHV- and EBV-associated germinotropic lymphoproliferative disorder. Blood.

[102] Wheat WH, Cool CD, Morimoto Y, Rai PR, Kirkpatrick CH, Lindenbaum BA, Bates CA, Ellison MC, Seris AE, Brown KK, Routes JM (2005). Possible role of human herpesvirus 8 in the lymphoproliferative disorders in common variable immunodeficiency. J Exp Med.

[103] Luppi M, Barozzi P, Marasca R, Savarino M, Torelli G (1996). HHV-8-associated primary cerebral B-cell lymphoma in HIV-negative patient after long term steroids. The Lancet.

[104] Kapelushnik J, Ariad S, Benharroch D, Landau D, Moser A, Delsol G, Brousset P (2001). Post renal transplantation human herpesvirus 8-associated lymphoproliferative disorder and Kaposi’s sarcoma. Br J Haematol.

[105] Matsushima AY, Strauchen JA, Lee G, Scigliano E, Hale EE, Weisse MT, Burstein D, Kamel O, Moore PS, Chang Y (1999). Posttransplantation plasmacytic proliferations related to Kaposi’s sarcoma-associated herpesvirus. Am J Surg Pathol.

[106] Luppi M, Barozzi P, Maiorana A, Artusi T, Trovato R, Marasca R, Savarino M, Ceccherini-Nelli L, Torelli G (1996). Human herpesvirus-8 DNA sequences in human immunodeficiency virus-negative angioimmunoblastic lymphadenopathy and benign lymphadenopathy with giant germinal center hyperplasia and increased vascularity. Blood.

[107] Chang Y, Cesarman E, Pessin MS, Lee F, Culpepper J, Knowles DM, Moore PS (1994). Identification of herpesvirus-like DNA sequences in AIDS-associated Kaposi’s sarcoma. Science.

[108] Oksenhendler E, Cazals-Hatem D, Schulz TF, Barateau V, Grollet L, Sheldon J, Clauvel JP, Sigaux F, Agbalika F (1998). Transient angiolymphoid hyperplasia and Kaposi’s sarcoma after primary infection with human herpesvirus 8 in a patient with human immunodeficiency virus infection. N Engl J Med.

[109] Wong SW, Bergquam EP, Swanson RM, Lee FW, Shiigi SM, Avery NA, Fanton JW, Axthelm MK (1999). Induction of B cell hyperplasia in simian immunodeficiency virus-infected Rhesus macaques with the simian homologue of Kaposi’s sarcoma-associated herpesvirus. J Exp Med.

[110] Luppi M, Torelli G (1996). Human herpesvirus 8 and interstitial pneumonitis in an HIV-negative patient. New Engl J Med.

[111] Muller A, Franzen C, Klussmann P, Wagner M, Diehl V, Fatkenhenen G, Salzberger B, Ablashi DV, Krueger GR (2000). Human herpesvirus type 8 in HIV infected patients with interstitial pneumonitis. J Infect Dis.

[112] Luppi M, Barozzi P, Schulz TF, Trovato R, Donelli A, Narni F, Sheldon J, Marasca R, Torelli G (2000). Non malignant disease associated with human herpesvirus 8 reactivation in patients who have undergone autologous peripheral blood stem cell transplantation. Blood.

[113] Luppi M, Barozzi P, Schulz TF, Setti G, Staskus K, Trovato R, Narni F, Donelli A, Maiorana A, Marasca R, Sandrini S, Torelli G (2000). Bone marrow failure associated with human herpesvirus 8 infection after transplantation. New Engl J Med.

[114] Luppi M, Trovato R, Barozzi P, Gibellini F, Potenza L, Riva G, Torelli G (2005). Suppressive effect of human herpesvirus-8/Kaposi sarcoma-associated herpesvirus on in vitro colony formation of hematopoietic progenitor cells. Leuk Res.

[115] Wu W, Vieira J, Fiore N, Banerjee P, Sieburg M, Rochford R, Harrington, Feuer G (2006). KSHV/HHV-8 infection of human hematopoietic progenitor (CD34+) cells: persistence of infection during hematopoiesis in vitro and in vivo. Blood.

[116] Abrams DI, Chinn EK, Lewis BJ, Volberding PA, Conant MA, Townsend RM (1984). Hematologic manifestations in homosexual men with Kaposi’s sarcoma. Am J Clin Pathol.

